# The garlic extract‐loaded nanoemulsion: Study of physicochemical, rheological, and antimicrobial properties and its application in mayonnaise

**DOI:** 10.1002/fsn3.3365

**Published:** 2023-06-09

**Authors:** Hamed Hassanzadeh, Mahshid Rahbari, Yaseen Galali, Mohamadyar Hosseini, Babak Ghanbarzadeh

**Affiliations:** ^1^ Department of Food Science and Technology, Faculty of Para‐veterinary Ilam University Ilam Iran; ^2^ Regional Research Group of Isfahan Standard Research Institute Isfahan Iran; ^3^ Food Technology Department, College of Agricultural Engineering Sciences Salahaddin University‐Erbil Erbil Iraq; ^4^ Department of Nutrition and Dietetics Cihan University‐Erbil Erbil Iraq; ^5^ Department of Food Science and Technology, Faculty of Agriculture University of Tabriz Tabriz Iran; ^6^ Department of Food Engineering, Faculty of Engineering Near East University Nicosia, Mersin Turkey

**Keywords:** antimicrobial activity, garlic extract, mayonnaise, organoleptic tests, rheology, water‐in‐oil nanoemulsion

## Abstract

In this research, garlic extract (GE)‐loaded water‐in‐oil nanoemulsion was used as a novel preservative and antioxidant in mayonnaise. GE (5%, 10%, 15%, and 25%) as a dispersed phase, olive oil as a continuous phase, and polyglycerol polyricinoleate (PGPR) as a low HLB surfactant, with a constant surfactant/garlic extract ratio (1:1), were used in the formulations of water‐in‐oil nanoemulsions. The properties of the active nanoemulsion, including droplet size, free radical scavenging capacity, antimicrobial activity against gram‐positive (*Staphylococcus aureus* [*25923 ATCC*]), and gram‐negative (*Escherichia coli H7 O157* [*700728 ATCC*]) were evaluated. The results showed that the mean droplet size of nanoemulsion increased from 62 to 302 nm and antioxidant capacity was also improved from 95.43% to 98.25% by increasing GE level from 5% to 25%. The minimum inhibitory concentration (MIC) and minimum bactericidal concentration (MBC) showed that antimicrobial activity against *S. aureus* could be observed only in high levels of GE (25%) in the formulation of nanoemulsion. The results of the total count analysis showed that the GE‐loaded nanoemulsion (NEGE) was effective against the microorganisms, particularly after 4 months of storage. The incorporation of GE and NEGE did not affect significantly the acidity of different mayonnaise samples; however, they affected the concentration of the primary product of lipid oxidation. Adding GE and NGE did not significantly affect the rheological properties of mayonnaise and all samples showed shear‐thinning behavior. Sensory evaluation showed that the samples with NEGE had higher scores in texture, spreadability, and mouthfeel, while the control samples had better scores in appearance, color, taste, and total acceptance. In general, the samples containing free GE (not encapsulated) had the lowest scores in all organoleptic characteristics.

## Graphical abstract

In this research, the garlic extract (GE)‐loaded nanoemulsions were used as a novel preservative and antioxidant in mayonnaise.

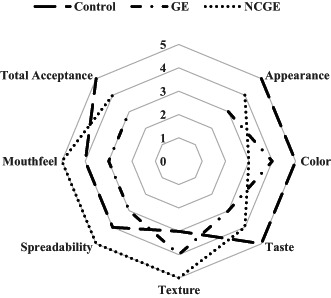

## 1 INTRODUCTION

Mayonnaise is oil‐in‐water emulsion salad dressing and it is widely consumed around the world. Mayonnaise is usually prepared from oil that is dispersed in an aqueous phase and consists of egg or egg yolk, vinegar, sugar, salt, spices (especially mustard), and some food additives, including stabilizers and preservatives. As there is no sufficient heat treatment in the mayonnaise preparation, preservatives are usually used in the formulation (Da Silva & Franco, 2012). The salts of organic acids, such as potassium sorbate (PS) and sodium benzoate (SB) are the most common preservatives that are used in mayonnaise formulation because of their antifungal and antimicrobial activities. However, some health adverse effects of synthetic preservatives have been reported by different researchers. Afshar et al. (2013) showed that 560 mg kg^−1^ of SB had reduced weight and crown‐rump length of the fetus of mice. Sohrabi et al. (2008) also reported that the SB at the content of 560 mg kg^−1^ decreased the weight of the ovaries and follicle‐stimulating and Luteinizing hormones and reduced progesterone hormone at the content of 280 mg compared with the control samples. Another study on lymph node cells isolated from mice treated with various levels of SB compared with control cells showed that SB can change the structure of lymphocytes and penetrate the cell membrane (Hu et al., 2008). Mamur et al. (2010) evaluated the genetic effects of PS on cultured and isolated human peripheral blood lymphocytes. Their results revealed the genotoxic effect of PS in cultured and isolated mammalian cells.

In recent years, due to possible risks and adverse effects of synthetic preservatives, using natural preservatives such as herbal extracts has received a great deal of attention. Furthermore, as mayonnaise is composed of 70%–80% oil, lipid oxidation is another reason for the quality deterioration in mayonnaise. Herbal extracts can have a high potential in reducing lipid oxidation due to their high content of phenolic compounds. Garlic contains a variety of biologically active compounds, including sulfur compounds, saponins, phenolic compounds, and polysaccharides. The main active components of garlic include its sulfur compounds, such as di‐allyl thiosulfonate (allicin), diallyl sulfide, diallyl disulfide, and diallyl trisulfide. Numerous studies have shown that garlic and its bioactive ingredients possess antioxidant, anti‐inflammatory, antibacterial, antifungal, immune system regulator, cardiovascular protection, anticancer, liver protection, gastrointestinal improvement, antidiabetes, anti‐obesity, protection of the body's nervous system, and kidneys protective properties (Shang et al., 2019).

Garlic extract has limitations for direct use in food products due to its high volatility and its effects on organoleptic properties. Therefore, it is better to encapsulate it and then incorporate it in food formulations. In recent years, many delivery systems including lipid‐based carriers and biopolymer‐based systems have been developed to encapsulate bioactive compounds in the food industry. One of the most common lipid‐based encapsulation systems is nanoemulsions that are prepared differently such as high‐ and low‐energy methods (Hassanzadeh et al., 2018).

Nanoemulsions are fine droplet emulsions that have a droplet size below ~200 nm and show relatively higher kinetic stability against flocculation, coalescence, and creaming than macroemulsions (conventional emulsion) due to their smaller droplet size (McClements, 2011; Saberi et al., 2013; Salvia‐Trujillo et al., 2015). Fabrication of nanoemulsions for encapsulation and controlled delivery of bioactive ingredients is one of the functional areas of nanotechnology in the food industry (Habibvand et al., 2022; Velikov & Pelan, 2008; Yang et al., 2012).

Rabbani et al. (2021) fabricated the phytosomal nanocarriers for encapsulation and delivery of resveratrol for application in mayonnaise and evaluated the physicochemical, microstructure, physical stability, free radical scavenging capacity, and encapsulation efficiency. Their results showed that phytosomal nanocarriers were able to keep the free radical scavenging capacity of resveratrol in mayonnaise during storage and could be considered in high‐fat foods to control the oxidation and maintain their nutritional characteristics. Savaghebi et al. (2021) fabricated the functional mayonnaise enriched by encapsulated *Sargassum boveanum* Algae extract in nano‐liposomes and reported that *S. boveanum* Algae extract in nano‐liposomes and BHT‐200 mayonnaises had significantly lower peroxide value and thiobarbituric acid‐reactive substances than the control and free *S. boveanum* Algae extract (*p* < .05). However, the color attributes, including lightness, redness, and yellowness of mayonnaise, were undesirably changed by the addition of free *S. boveanum* Algae extract.

de Souza Mesquita et al. (2020) formulated mayonnaise as a model food for increasing the accessibility of carotenoids from *Bactris gasipaes* fruits. Carotenoids from *B. gasipaes* fruits were prepared by ultrasound using sunflower oil to prepare an oil‐in‐water food emulsion similar to typical mayonnaise with a reduced fat percent. Their results present a pleasant idea for carotenoid incorporation into new foodstuffs, representing a new model to design functional products with more bioavailable lipophile bioactive components. This design can be applied to improve the functionality of food systems with insufficient nutritional properties. Therefore, the aims of the present study were to produce the GE‐loaded water‐in‐oil nanoemulsion (NEGE) and then using of NEGE in mayonnaises as a preservative, antioxidant, and functional nutritional agents.

## 2 MATERIALS AND METHODS

### 2.1 Preparation of garlic extract

First, the garlic cloves were soaked in water for better separation of the skin, and then the skin and bottom of the garlic cloves were removed and the garlic cloves were washed. After washing, the garlic was poured into a mixer, and after crushing it completely, it is mixed with distilled water at a ratio of 1:1. The mixture was then stored at a temperature below 15°C for 24–48 h. Finally, the mixture was passed through a cloth strainer to separate the coarse garlic particles and the filtered liquid was passed through a filter paper. Finally, the extract was centrifuged at a rotational speed of 4248 *g* for 15 min, and thus the garlic extract was obtained.

### 2.2 Preparation of nanoemulsions

After preparing the garlic extract, the olive oil was mixed with the PGPR surfactant at a ratio of 1:1 with dispersed phase (GE) while stirring. Garlic extract was then added dropwise and slowly to the stirring oil and surfactant mixture at a rotational speed of 29.5 *g*. The prepared mixture was continued to mix at a rotational speed of 57.82 *g* for 20 min and transferred to an ultrasonic homogenizer for further homogenization and the desired nanoemulsions were prepared (Mohammed et al., 2021).

### 2.3 Droplet size distribution analysis

To measure particles, all of the nanoemulsions are diluted at a ratio of 1:50 in oil to prevent multiple particle scattering. The mean particle size (*Z*_average_) and polydispersity index (PDI) were determined by dynamic light scattering (Malvern Instrument) at the temperature of 25°C and an angle of 90°.

### 2.4 Free radical scavenging capacity

The antioxidant properties of the prepared nanoemulsions and garlic extract were measured using DPPH method. In this method, 4 mL of 60 μM free radical methanol solution of DPPH was mixed with 0.2 mL of the prepared nanoemulsion and kept at room temperature for 60 min. Then, the absorbance of the solution was read at 517 nm using a spectrophotometer. Also, 4 mL of DPPH with a sample containing 0.2 mL of methanol was determined as a control sample, and radical scavenging capacity was calculated using the relevant below formula (Equation 1) (Hasanzadeh et al., 2017).

(1) $$\text{Free radical scavenging}=\frac{\text{Control absorbance}-\text{Sample absorbance}}{\text{Control absorbance}}\times 100$$

### 2.5 The minimum inhibitory concentration and minimum bactericidal concentration

Minimum inhibitory concentration (MIC) and minimum bactericidal concentration (MBC) determinations were performed by the microdilution method. For this purpose, the bacteria culture was freshly prepared. Firstly, the selected bacteria, including *Staphylococcus aureus* (*25923 ATCC*) and *Escherichia coli H7 O157* (*700728 ATCC*) were cultured on Mueller‐Hinton broth and incubated for 24 h at 37°C. So, to obtain bacteria sediment, Falcon tubes were centrifuged for 15 min at around 1062 *g*. Sediment bacteria were washed twice with sterile saline and in the fourth stage of the centrifuge tube; a dilution of 0.5 McFarland was provided by standard McFarland tubes and sterile saline. TSB culture medium was prepared separately and 100 μL of it was poured into 7‐well plates. TBS culture was separately prepared and various dilutions of nanoemulsions in this medium in a 96‐well plate were prepared. Firstly, 100 μL of nanoemulsion with 100 μL TBS culture were mixed and after stirring, 100 μL of this mixture was transferred to the next wells and diluted by 100 μL of TBS culture again, and this was repeated five times. And so, six different dilutions of nanoemulsion were prepared in 6 wells of the plate, and the seventh was considered as the control without the nanoemulsion. 1/2–1/64 dilutions of the nanoemulsions were formed. Next, 20 μL of prepared dilution of bacteria were inoculated in 6 wells containing culture medium and nanoemulsions. In the seventh well, only 100 μL of culture medium was added and considered a control. The prepared dilution of bacteria was equal to 0.5 McFarland (approximately contain 1.5 × 10^8^ CFU mL^−1^), but to obtain more accurate results, 0.5 McFarland was diluted by sterile saline to achieve approximately 5 × 10^5^ CFU mL^−1^. Finally, 96‐well plates were placed at 37°C for 18 h and then MIC was determined by comparing the turbidity of treated wells with control wells. To determine the MBC, from bacteria treated 96‐well plate, 1 dilution lower than MIC dilution and 2 dilutions higher than it, were cultured linearly on Mueller‐Hinton agar medium. The lowest concentration, at which the line growth was not found on agar, was considered MBC (Andrews, 2001).

### 2.6 Preparation of mayonnaise

The mayonnaise samples were prepared according to the basic formulation of low‐fat mayonnaise which is now produced commercially. The control mayonnaise recipe contained the following ingredients based on the percentage (w/w): vegetable oil 40, egg yolk 9, and vinegar 9 (11% w/v acetic acid), salt 1, sugar 5, mustard 0.3, stabilizer 0.15, modified starch 2, and water 42.55. The mayonnaise samples were prepared using nanoemulsion as disperse phase or garlic extract as the aqueous phase. Also, the amounts of preservatives including SB and PS varied ranged from 0, 375, and 750 ppm according to the experimental design. The mixtures composition in the mayonnaise formulation is shown in Table 1. For mayonnaise preparation, a standard mixer was used (Moulinex, HM312). First, the modified starch and stabilizers were dissolved in an amount of oil separately. Then dissolved starch and dry matters were mixed followed by the addition of the dissolved stabilizers and about one‐third of water. Then a small amount of the oil was added. The ingredients were blended for approximately for 5 min. Afterward, one‐third of vinegar was slowly added to the mixer. Finally, the remaining oil, water, and vinegar were gradually added and admixed in a mixer. The prepared mayonnaise was transferred to glass bottles and stored at room temperature (25°C) for further analysis.

**TABLE 1:** Mixtures composition in the mayonnaise formulated with nanoemulsion, garlic extract, and preservative.

Runs	Preservative concentration (ppm)	Nanoemulsion (NE)/none/garlic extract (GE)
1	375	None
2	750	NE
3	0	None
4	375	GE
5	750	GE
6	375	NE
7	750	None
8	0	GE
9	375	NE
10	750	GE
11	0	None
12	0	NE

### 2.7 Microbial assay

Total plate count, acid‐resistant bacteria, *E. coli* bacteria, mold, and yeast of mayonnaise samples were determined according to ISO international standards. These assessments were done monthly throughout the 4 months of storage at 25°C.

### 2.8 Acidity measurement

About 15 g of the prepared mayonnaise was mixed with 200 mL of distilled water, then 3–4 drops of phenolphthalein was added and titrated with NaOH 0.1 N until the first color changed to pink, and finally the acidity value calculated by the corresponding formula (Tavakoli et al., 2021).

### 2.9 Oxidation stability (peroxide value)

IDF standard method was used for determining the peroxide value of all samples. To determine the peroxide value, 8.9 mL methanol–chloroform (3 + 7) (V/V) was mixed in a glass test tube using a mixer for 2–4 s and then 50 μL of ammonium thiocyanate solution was added and then samples were taken for 2–4 s and mixed with a vortex mixer. Finally, 50 μL ferrous sulfate (II) was added and the samples were mixed for 2–4 s with a vortex mixer.

After 5 min of incubation at room temperature, absorbance versus blank (all indicators except sample) were read by spectrophotometer, and then peroxide value was calculated based on the following equation (Equation 2). The whole procedure is performed in the dark for 10 min.

(2) $$\text{Peroxide value}=\frac{\left(A_{s}-A_{b}\right)\times m}{55.84\times m_{0}\times 2}$$

where *A*_s_ = sample absorbance; *A*_b_ = Blank absorbance; *m* = slope, obtained from the calibration curve, *m*_0_ was the sample weight and 55.84 was the atomic weight of iron.

### 2.10 Steady and oscillatory shear rheological analysis

Flow behavior and oscillatory tests were performed using a rheometer (Anton Paar Physica MCR300). Flow properties of the mayonnaise samples were determined at 20°C using a parallel stainless steel plate with 20 mm diameter in the shear rate range of 0.1–100 s^−1^. To determine the flow behavior, the experimental data were fitted to a Power‐law equation (Equation 3):

(3) $$\tau =K{\dot{\gamma }}^{n}$$

where *τ* is the shear stress (Pa), γ is the shear rate (1/s), *K* is the consistency index (Pa.sn), and *n* is the flow behavior index.

The dynamic oscillatory tests were conducted over a frequency range of 0.1–50 Hz at a constant strain of 0.5% (within the linear viscoelasticity range that was previously established by the strain sweep tests). Data were collected and rheological parameters were calculated using a rheometer software program. Storage modulus (G′) and loss modulus (G″) and tan (delta) versus frequency was measured for all the samples.

### 2.11 Sensory evaluation

After 1‐day of storage at room temperature, the organoleptic tests were done for the mayonnaise samples. The mixed 5‐point hedonic scale was used (scale, 1 = the least acceptable; 5 = the most acceptable). The texture, appearance, odor, taste, and overall acceptance were measured by 12 panelists. Some bread and a cup of water were provided between samples to clean the palate.

### 2.12 Statistical analysis

In the first stage of this study (fabrication of nanoemulsions), experiments were performed in duplicate at least for calculating the mean and standard deviation. One‐way ANOVA and Tukey tests were used for the analysis of data at (*α* = 0.05) using Minitab software (version 17) for mean treatments comparison. D‐optimal design (preservative level in tree level and types of enriched mayonnaises) was used by Design‐Expert software (version 10) for analyzing mayonnaises in the second stage. Table 1 shows mayonnaise formulated with nanoemulsion, garlic extract, and preservatives.

## 3 RESULTS AND DISCUSSION

### 3.1 Droplet size analysis

The results of DL for the GE‐loaded water‐in‐oil nanoemulsions showed that the particle size was increased significantly with increasing volume fraction (garlic extract). As shown in Table 2, by increasing the percentage of GE from 5% to 25%, the particle size has increased from 62 to 302 nm. This indicates that the particle size of water‐in‐oil nanoemulsions is affected by the volume fraction. This could be attributed to the increase in water droplet collisions with increasing volume fraction which probably led to the merger of them in the emulsion. Despite the same ratio of surfactant and dispersed phase, the surfactant's ability to surround the dispersed phase at higher concentrations was decreased due to the congestion of surfactant and dispersed phase (GEO) molecules. In accordance with these results, Peng et al. (2010) optimized water‐in‐oil nanoemulsions with a mixture of surfactants and reported that by keeping the surfactant ratio constant (10%) and increasing the water content in water‐in‐oil nanoemulsion formulations from 10% to 40%, their particle size increased from 13 to 186 nm. Also, Porras et al. (2005) studied the production of water‐in‐oil nanoemulsions and reported that the particle size was increased from 30 to 120 nm by increasing the aqueous phase ratio from 5% to 25%. Also, the average droplet size between 132 ± 2.0 and 145 ± 1.0 nm was obtained in O/W pepper nanoemulsions produced by high‐pressure homogenization with an inverse ratio between particle size and dispersed phase ratio (Galvão et al., 2018).

**TABLE 2:** Particle size obtained from DLS for water‐in‐oil nanoemulsions.

Type of nanoemulsion	Particle size (nm)
Nanoemulsion containing 5% GE	62.4 ± 5
Nanoemulsion containing 15% GE	197.6 ± 12
Nanoemulsion containing 25% GE	302 ± 20

### 3.2 Free radical scavenging capacity

**FIGURE 1:**
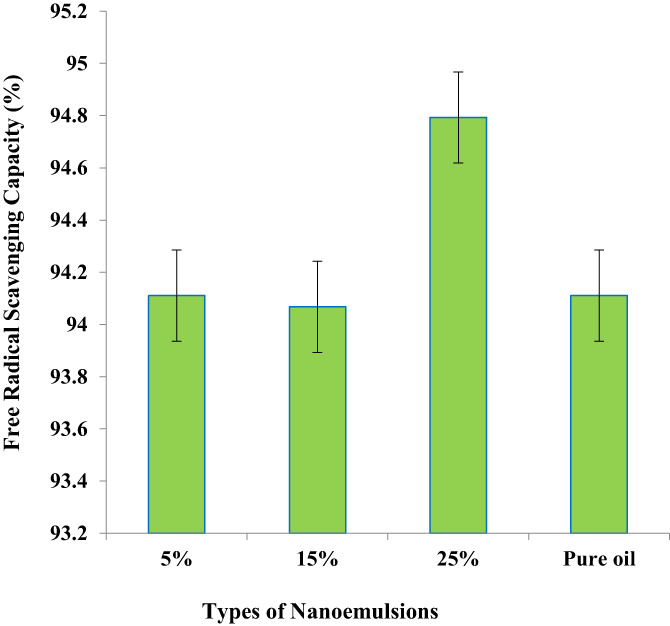
Free radical scavenging capacity of water‐in‐oil nanoemulsions prepared by garlic extract and olive oil.

The results obtained from the DPPH method for water‐in‐oil nanoemulsions showed that in general, this type of nanoemulsion has more free radical scavenging capacity than oil‐in‐water nanoemulsions. This can be due to the fact that there is some phenolic compounds in the aqueous phase of water–oil nanoemulsions and the antioxidant ability of the oil itself (Hassanzadeh et al., 2018). As can be seen in Figure 1, there is a significant differences between the prepared nanoemulsions with different formulations and all the prepared formulations have more than 90% free radical scavenging capacity. The antioxidant capacity was enhanced from 94.1% to 94.7% by increasing the GE level from 5% to 25%. This is might be attributed to the presence of high‐content polyphenol components in GE.

### 3.3 MIC and MBC of nanoemulsions

Although to our best knowledge limited research has been published on the antimicrobial properties of water‐in‐oil nanoemulsions, in this study, the antimicrobial properties of water‐in‐oil nanoemulsions containing GE have been investigated. The MIC and MBC of water‐in‐oil nanoemulsions containing different percentages of the extract showed that only high percentages of GE in the formulation showed antimicrobial activity against *S. aureus* (Table 3).

**TABLE 3:** Minimum inhibitory concentration and minimum bactericidal concentration of nanoemulsions by percentage for the two tested bacteria.

Nanoemulsion type		Nanoemulsion containing 5% garlic extract		Nanoemulsion containing 15% garlic extract		Nanoemulsion containing 25% garlic extract
Bacterial type	*E. coli*	*S. aureus*	*E. coli*	*S. aureus*	*E. coli*	*S. aureus*
MIC	–	–	–	–	–	50
MBC	–	–	–	–	–	–

By comparing the antimicrobial activity of oil‐in‐water nanoemulsions (Hassanzadeh et al., 2018) and water‐in‐oil nanoemulsions, it can be observed that water‐in‐oil nanoemulsions have lower antimicrobial activity. This could be due to the higher intensity of volatiles in garlic essential oil than in its extract. In a similar pattern with oil‐in‐water nanoemulsions, it was found that they have more antimicrobial activity against gram‐positive bacteria than gram‐negative bacteria (Hassanzadeh et al., 2022). Similar results were reported by Hassanzadeh et al. (2019) for water‐in‐oil nanoemulsions containing GE by disk diffusion method via the diameter of the bacterial growth inhibition zone (Hassanzadeh et al., 2019).

Some distinctive mechanisms have been proposed to describe the antimicrobial properties of garlic. Inhibition of DNA, RNA, and cell protein synthesis is one of the proposed mechanisms (Feldberg et al., 1988). It is stated that a major part of the antimicrobial properties of garlic is related to allicin and its metabolites. These compounds exert their antimicrobial activity through specific inhibition of the enzyme acetyl‐coenzyme A‐synthetase. Inhibition of this enzyme inhibits the biosynthesis of lipids and fatty acids and ultimately impairs cell viability.

One of the characteristic properties of garlic organosulfur compounds is their ability to penetrate through membrane phospholipids. Among the compounds in aqueous garlic extract, diallyl thiosulfate is the most effective compound in the biological and antimicrobial activities of garlic. As shown in Table 3, due to the encapsulation of most of the volatile and functional compounds of GE during the emulsification process, nanoemulsions with a lower percentage of GE did not show antimicrobial activity, and only nanoemulsions containing 25% GE had antimicrobial activity against *S. aureus*.

### 3.4 Mayonnaises tests

#### 3.4.1 Microbial characteristics

**FIGURE 2:**
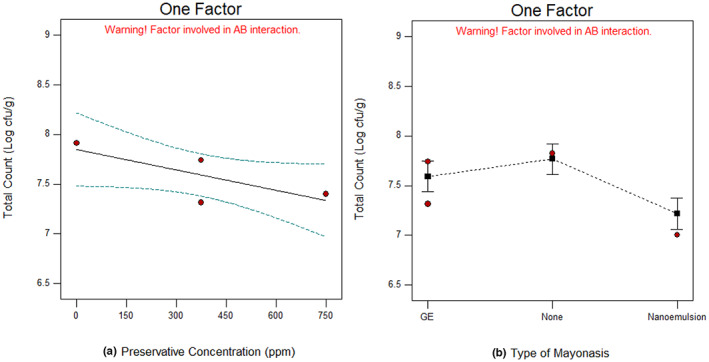
Total count of mayonnaise after 4 months, at different preservative concentrations (a), and mayonnaise formulated with garlic extract, nanoemulsion, or none (b).

The results of the total count showed that the studied factors have been effective against a number of microorganisms, particularly after 4 months of storage. SB, as a preservative, has played the most important role in reducing total count. The addition of GE as well as nanoemulsions containing GE also reduced the total count (Figure 2). As discussed in the previous part, GE can reduce the microbial load of mayonnaise due to its organosulfur compounds such as allicin and similar compounds. However, the nanoemulsions containing GE may be due to the slow and continuous release of volatile compounds that shows higher effects on the total count.

Mansouri et al. (2021) evaluated the antibacterial properties of nanoemulsion containing *Thymus daenensis* L. essential oil in mayonnaise and reported that the non‐encapsulated essential oil showed more antibacterial properties compared with the optimal nanoemulsion. The bacterial control in mayonnaise was approximately equal to SB (in maximum limit 1 g kg^−1^) and the optimal nanoemulsion (½ MIC) for 24 h. Savaghebi et al. (2021) fabricated the functional mayonnaise enriched with *Sargassum boveanum* algae extract in nano‐liposomes (BAE‐NLs) and they found that total viable and fungal counts of the samples incorporated with BAE‐NLs and SB (1000 mg kg^−1^) showed significantly lower colonies compared with the control samples and samples containing free BAE after storage.

Gorjian et al. (2021) compared the effect of nanoliposome and nanoniosome containing myrtle extract as a natural preservative and SB on microbial characteristics of mayonnaise. These results showed that the counts of *E. coli* and hetero‐fermentative *lactobacilli* were negative for all treatments and similar to standard. The microbial growth of the mayonnaise containing the nanoliposome and nanoniosome had a significant effect (*p* < 0.05) on controlling the mold, yeast, and acid‐resistant bacteria in comparison with the control sample during the storage time.

Rafiee et al. (2018) produced mayonnaise enriched by nanoliposomes containing pistachio green hull's phenolic compounds as natural biopreservatives. The results showed that nanoliposomes with 1000 mg  kg^−1^ of phenolic components had the highest inhibition properties on total viable and fungal counts. After 4 months of storage, the microbial count in samples treated with phenolic compounds similar to the samples containing 1000 mg kg^−1^ of SB are within the permissible level.

In a similar research, Chaudhari et al. (2020) evaluated the preservative potential of anethole‐based chitosan nanoemulsion (Ant‐eCsNe) to control deterioration of stored maize samples from fungal infestation, aflatoxin B1 (AFB1) contamination, and lipid oxidation. Their results revealed that Ant‐eCsNe holds good potential to be applied as a food preservative to reduce fungal and aflatoxin contamination causing deterioration of stored maize.

#### 3.4.2 Acidity measurement

**FIGURE 3:**
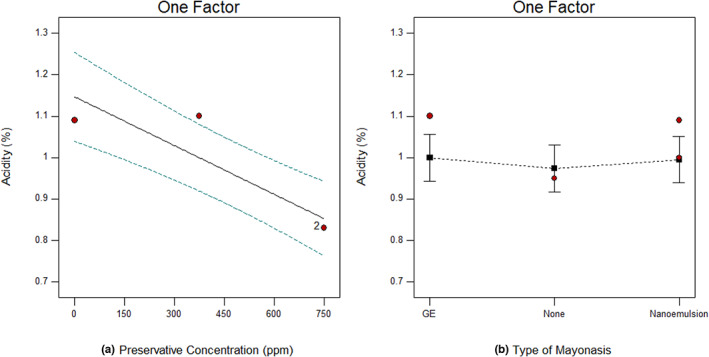
Acidity of mayonnaise at different preservative concentration (a), and mayonnaise formulated with garlic extract, nanoemulsion or none (b).

Acidity is one of the most important properties of mayonnaise, dressings, and sauces for recognizing the growth and survival of pathogenic bacteria (Tavakoli et al., 2021). Acetic acid is the main acid in mayonnaise, which presents as various kinds of vinegar. The effects of acidity on microorganisms are as follows: (1) the impact of pH alone, (2) the effect of undissociated forms of a special acid, and (3) the particular effects of organic acids (Jalilzadeh et al., 2018). As shown in Figure 3, statistical analysis of data demonstrated that there is no significant difference between the acidity of the differently formulated mayonnaises. This means that the addition of GE and nanoemulsions containing GE did not affect the acidity (*p* > 0.05). In contrast, the addition of SB significantly affected the acidity of mayonnaises and decreased from 1.15 to 0.85 by changing SB from 0 to 750 ppm (*p* < 0.05). Similar results were recorded by Tavakoli et al. (2021) as they reported the higher pH value in mayonnaise samples containing preservatives compared with the control sample during storage.

Gorjian et al. (2021) compared the effect of nanoliposome and nanoniosome containing myrtle extract as a natural preservative and SB on microbial, physicochemical, and organoleptic characteristics of mayonnaise and concluded that the highest pH (4.2) was seen in sauce sample containing SB.

#### 3.4.3 Peroxide value

**FIGURE 4:**
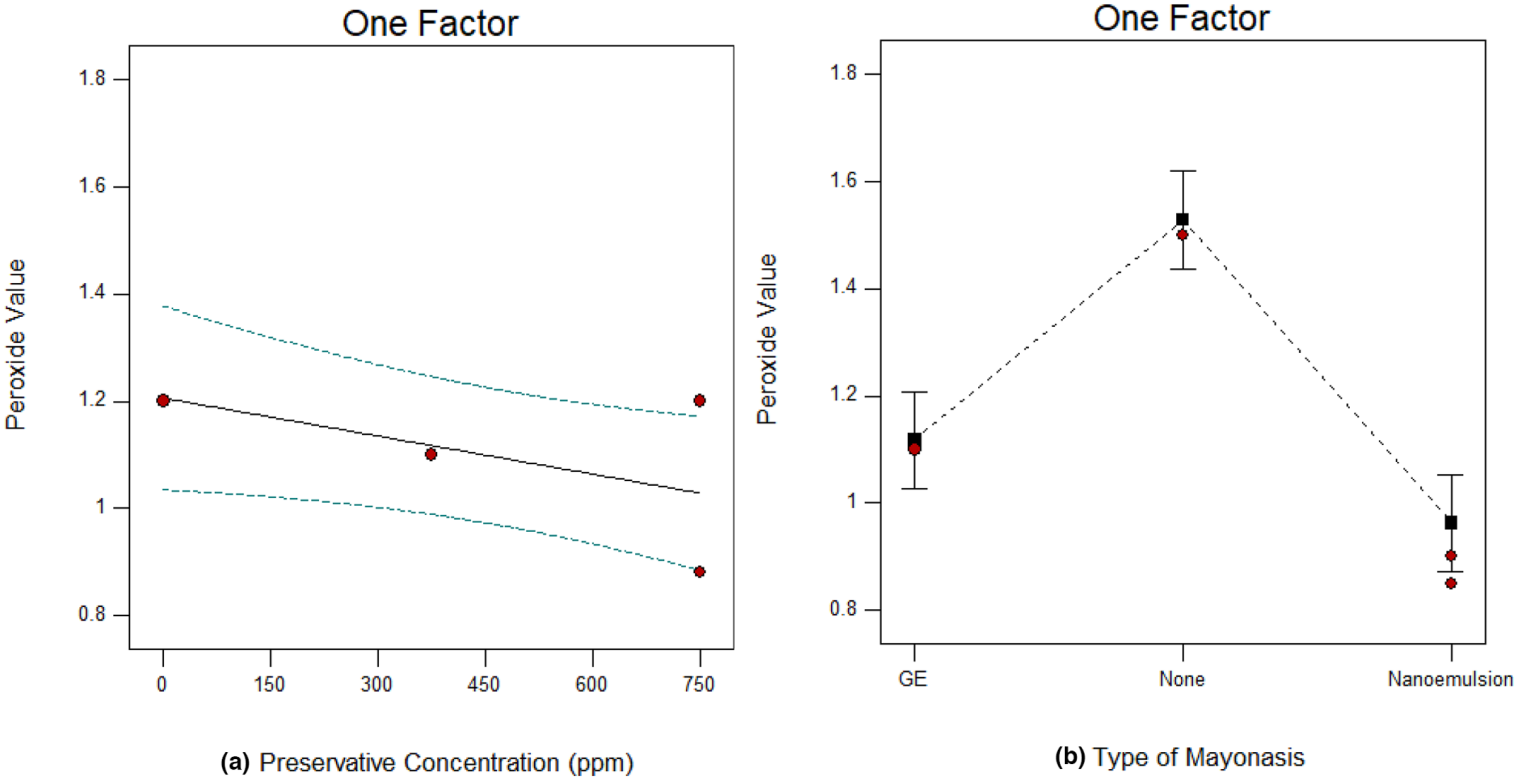
Peroxide value of mayonnaise at different preservative concentrations (a), and mayonnaise formulated with garlic extract, nanoemulsion, or none (b).

Peroxide index is a major criterion for measuring hydroperoxides which are the primary products of lipid oxidation. Hydroperoxides were increased and then decreased during oxidation, and this process continues due to their successive formation and degradation [19]. Since mayonnaise is an oil‐in‐water emulsion and its oily phase which is in contact with a large area of water, it is very prone to oxidative damage. Therefore, an increasing trend in the number of peroxides due to the intensification of oxidation can be observed for sauce samples in particular during long storages. As can be seen in Figure 4, statistical analysis of study factors on peroxide value declared that preservative concentration and addition of GE and nanoemulsions containing GE affected the primary lipid oxidation. Meanwhile, the addition of GE and nanoemulsion containing GE showed a greater effect on reducing the peroxide index. The lowest peroxide index (<1) is observed when nanoemulsions containing GE are used in mayonnaise formulation. The addition of GE can reduce the primary products of oxidation and reduce the peroxide index due to its high polyphenolic content and neutralization of free radicals during oxidation. In similar research, the peroxide value was decreased by raising SB and using nanoliposomes and nanoniosomes containing case leaf extract (Gorjian et al., 2021). Also, Rabbani et al. (2021) fabricated phytosomal nanocarriers for encapsulation and delivery of resveratrol and reported that the results showed that phytosomal resveratrol was able to protect the antioxidant properties (free radical scavenging capacity) of resveratrol in mayonnaise during storage time and could be used in high‐fat foods to minimize lipid oxidation and improve their oxidative stability and nutritional properties. In recognition of this, Berenji et al. (2021) fabricated resveratrol food‐grade lipid nanocarriers as a potential antioxidant in mayonnaise and concluded that the peroxide value in the sample containing resveratrol–lipid nanocarriers reduced slightly and antioxidant activity was higher than other samples. In general, the use of resveratrol as natural antioxidants prevents lipid oxidation in mayonnaise during storage, and lipid nanocarriers can be a suitable system for encapsulating resveratrol to preserve its antioxidant properties. In another research, corn oil designer lipid‐based mayonnaise showed considerable oxidative stability compared with the control samples based on recorded data from acid value, peroxide value, p‐anisidine value, and TOTOX value (Jadhav et al., 2022).

#### 3.4.4 Rheological analysis

##### Steady‐state shear rheology

**FIGURE 5:**
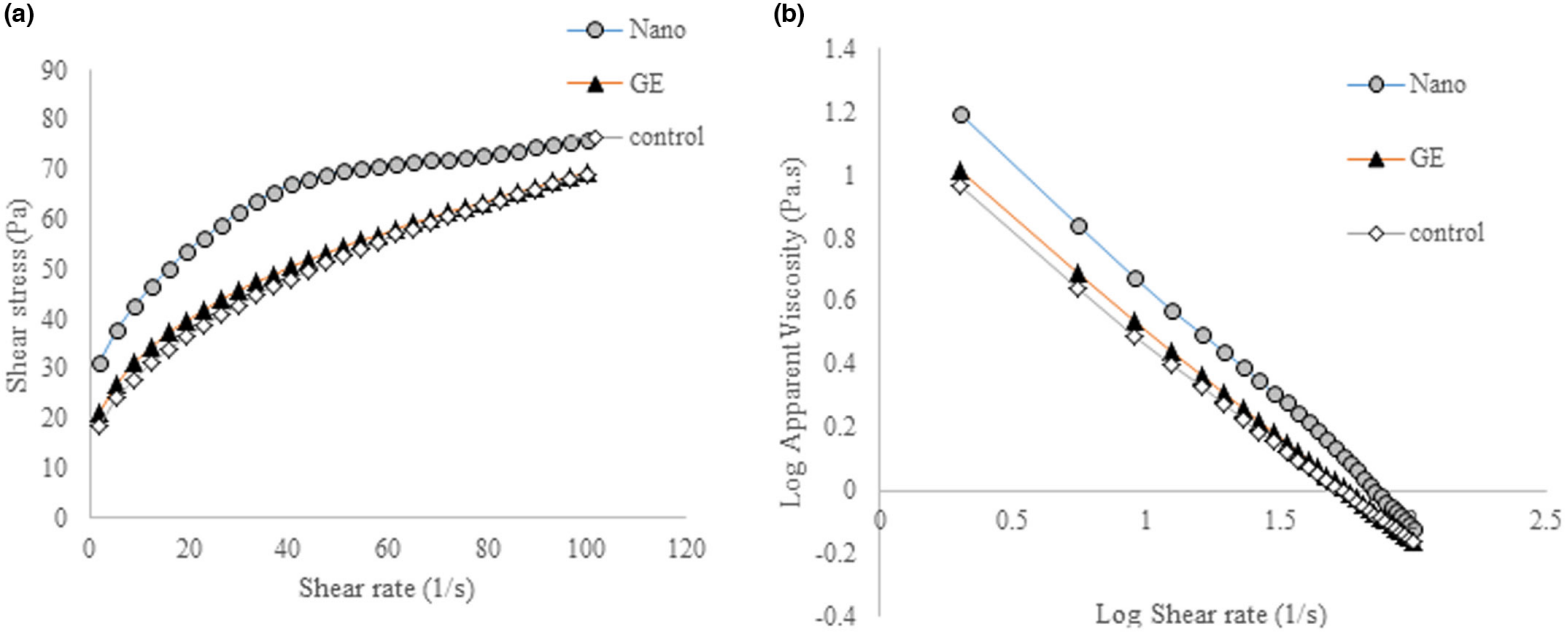
Flow curves of the mayonnaises (a), and apparent viscosity versus shear rate for the mayonnaise samples (b).

Figure 5a shows the relationship between the shear stress and shear rate of mayonnaise samples. It can be seen that the relationship between shear stress and shear rate is nonlinear. So, mayonnaise samples are classified as non‐Newtonian fluids. On the other hand, due to the decrease in apparent viscosity with increasing shear rate, mayonnaise samples have a shear thinning (Pseudoplastic) behavior. The relationship between the apparent viscosity and shear rate of mayonnaise samples is shown in Figure 5b. As the shear rate increased, the apparent viscosity of the samples decreased, and they showed a thinning behavior with the shear. In a concentrated emulsion, droplet cohesion leads to the formation of a three‐dimensional network of aggregated droplets. Applying a shear force on emulsions changes the shape of the droplets from spherical to elliptical and reduces the friction between the fluid and the droplets. At higher shear forces, the accumulated droplets are progressively separated and isolated and the emulsion resistance to flow (apparent viscosity) is reduced.

As can be seen, the mayonnaise sample containing nanoemulsion showed higher apparent viscosity than the one containing garlic extract and control, in the range of applied shear rates. It seems that the presence of PGPR in the formation of water‐in‐oil nanoemulsion, with its emulsifying and stabilizing properties and increasing the oil phase of mayonnaise, reduces the movement of oil droplets, and stabilizes the mayonnaise as an oil‐in‐water emulsion.

Apparent viscosity data at intermediate shear rates (20–80 1/s) are used to study sensory evaluation and mouthfeel of fluid food materials. For this purpose, the apparent viscosity of the samples at 50 s^−1^ shear rate was studied (Table 4). The results showed that the treatment containing nanoemulsion had the highest apparent viscosity. The control sample had the lowest value, meanwhile, the viscosity of the sample containing GE is close to the control sample which can be justified according to the nature of the extract and its participation in the continuous phase of mayonnaise and the increase of the continuous phase. In a previous similar study, the use of water‐in‐oil nanoemulsion in the mayonnaise formulation increased the volume fraction of the dispersed phase (Oil) that led to an increase in viscosity (Ayatollah Mousavi et al., 2008).

**TABLE 4:** Power‐law model parameters and apparent viscosity for mayonnaise samples.

Samples	*n*	*K* (Pa.s)	*R* ^2^	Apparent viscosity at 50 s^−1^ (Pa.s)
Mayonnaise with NGE	0.237	24.61	0.98	1.37
Mayonnaise with free GE	0.316	15.72	0.996	1.07
Control mayonnaise	0.355	13.065	0.993	1.03

The power‐law model is widely used to describe the relationship between shear stress and shear rate in many oil‐based food products and emulsions (Rahbari et al., 2015). The shear stress data‐shear rate data of different mayonnaise samples were fitted in power‐law model with high coefficient of determination (*R* ^2^). Table 4 shows the values of the power‐law model parameters for each of the mayonnaise samples. In general, the samples containing nanoemulsion showed the lowest flow behavior index value but the highest consistency index value. The role of water‐in‐oil nanoemulsion in increasing the dispersed phase content of mayonnaise (as oil‐in‐water nanoemulsion) can be considered as the reason for this change in flow behavior.

##### Dynamic (oscillatory) rheological behavior

**FIGURE 6:**
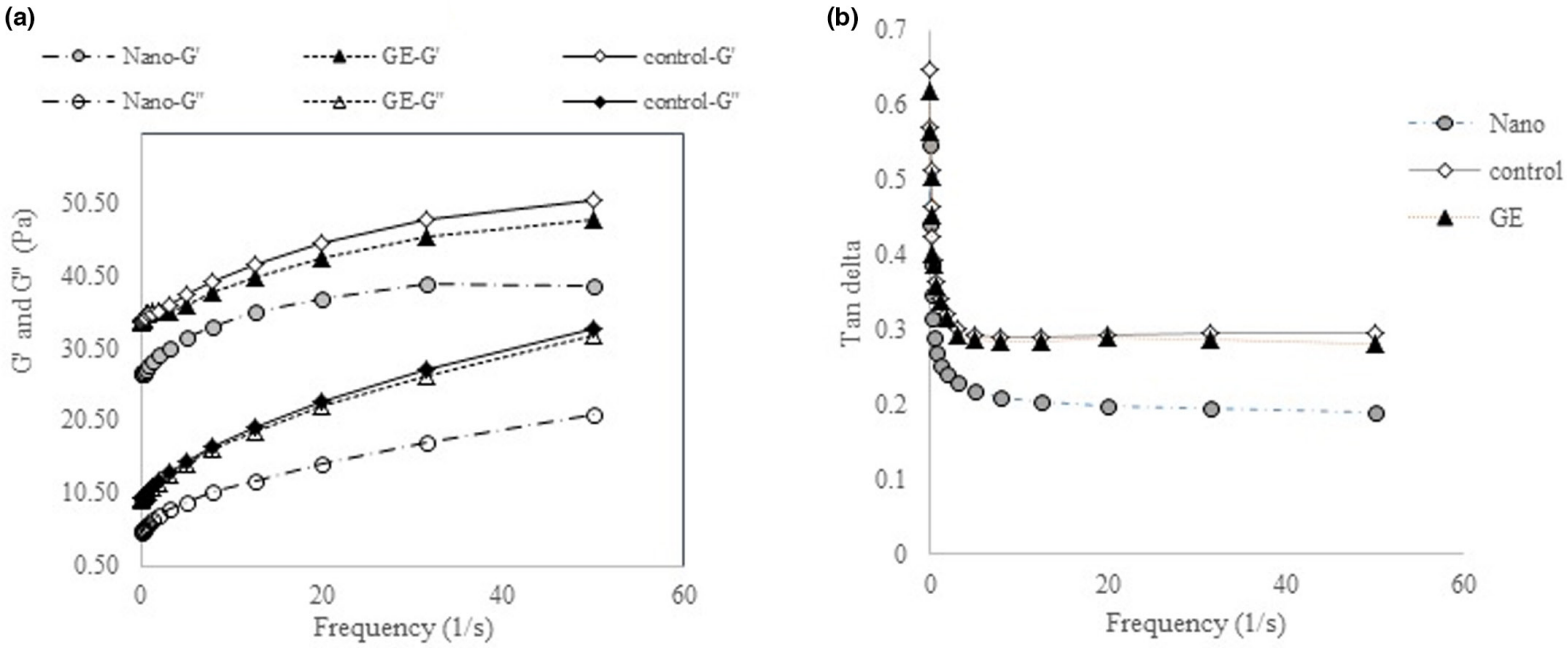
Dynamic mechanical spectra of mayonnaise samples G′ and G′′ (a), and tan (delta) vs. frequency (b).

In the frequency sweep test, a gradual increase in storage modulus and loss modulus of treatments was observed with increasing frequency (Figure 6). The storage modulus is an indication of the amount of elastic behavior and the amount of energy recovered per unit volume per complete cycle of the strain wave. Furthermore, the loss modulus or viscosity modulus (G″) is an indication of the amount of flow behavior and the amount of energy lost per unit volume per complete cycle of strain wave. In the frequency sweep test, if G″ < G′, the sample shows solid viscoelastic behavior, and if G″ > G′, the sample shows liquid viscoelastic one. In this research, the storage modulus data were higher than the loss modulus in all frequencies which shows weak gel behavior. Various studies have also reported that mayonnaise at frequencies of 0.10–1 Hz shows weak gel properties (Bernardi et al., 2011). The ratio of G″ to G′ indicates another parameter called the loss tangent. If the loss tangent is >1, this means that the viscoelastic material is liquid, and <1 indicates the solid viscoelastic behavior in the material. The loss tangent of mayonnaise samples at 1 Hz is presented in Figure 6b. The results showed that in all samples, tan *δ* was <1, which indicates the higher elastic property than viscose and consequently, the solid viscoelastic behavior of the samples.

Other researchers have observed similar results in preparing different types of low‐fat mayonnaise using different levels of gums, such as xanthan and guar, or using modified starch (Bajaj et al., 2019; Su et al., 2010). Among the samples, the lowest loss tangent was observed in mayonnaise enriched with NEGE, which indicates that this sample had more elastic properties. The highest amount of tan δ was observed in the control treatment, which indicates a greater tendency of this sample to quasi‐liquid behavior. A higher aqueous phase in formulations containing GE and control samples may increase the free water of these formulations and the tendency of these samples to quasi‐liquid behavior (Figure 6). Khan et al. (2020) developed encapsulated vitamin D‐fortified mayonnaise (VDFM) using whey protein isolates (WPI) and soy protein isolates (SPI) as encapsulating materials. They reported VDFM showed pseudoplastic behavior, along with significant effects on the loss modulus. The power‐law model was used for the flow characteristics of VDFM. The maximum scores for oscillatory measurements of mayonnaise samples were related to mayonnaise formulated with WPI, while the least score was shown by mayonnaise formulated with SPI.

### 3.5 Sensory evaluation

**FIGURE 7:**
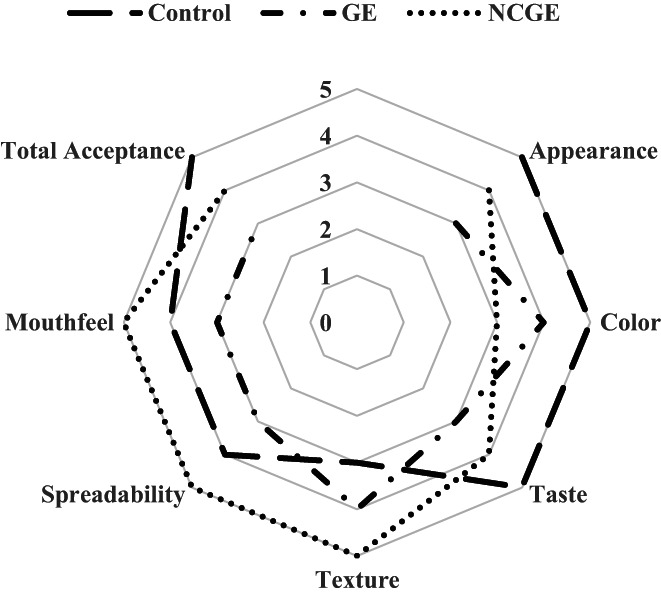
Spider plot for describing the sensory properties of mayonnaise formulated with garlic extract (GE), nanoemulsion (NEGE), and control.

Results from organoleptic characteristics (appearance, color, taste, texture, spreadability, mouthfeel, and total acceptance) that were evaluated by panelists showed that samples containing nanoemulsions of GE had higher scores in texture, spreadability, and mouthfeel while the control samples had better scores in appearance, color, taste, and total acceptance. In general, samples containing GE (not encapsulated) had the lowest scores in all organoleptic characteristics (Figure 7). Mansouri et al. (2021) studied the antibacterial activity and stability of nanoemulsion containing *T. daenensis* L. essential oil in mayonnaise. Their optimal nanoemulsion samples obtained significantly (*p* < 0.05) higher organoleptic scores (appearance, taste, and mouthfeel) than mayonnaise with pure essential oil.

In a similar attempt, Flamminii et al. (2020) measured the organoleptic characteristics of mayonnaise fortified with encapsulated olive leaf phenolic extracts and reported that the sensory evaluation enriched mayonnaise samples, particularly the mayonnaise enriched with OLE‐loaded microcapsule obtained the lowest total acceptance score. Also, the fortified mayonnaise samples had a lower spreadability and a higher salty and bitter sense, leading to decreased total acceptance. Khan et al. (2020) fabricated the mayonnaise enriched with vitamin D encapsulated in protein‐based carriers and evaluated their bioavailability, rheology, and sensory evaluation. They reported that the highest value for overall acceptability was acquired by 5:5%WPI: SPI‐encapsulates, thus proceeding for in vivo trials. In addition, de Souza Mesquita et al. (2020) designed mayonnaise as a model food for improving the bioaccessibility of carotenoids from *Bactris gasipaes* fruits and reported that the food model developed in their study had high total acceptance in all the measured characteristics.

## 4 CONCLUSION

Recently, nanocarrier emulsions containing extracts and essential oils are known as biopreservative in food formulation. Water‐in‐oil nanoemulsions containing garlic extract showed an acceptable antimicrobial and antioxidant capacity for food applicants. The addition of garlic extract as well as nanoemulsions containing garlic extract also had an effect on reducing total count and peroxide value which led to increased oxidative stability and shelf life. In terms of both total count and peroxide value, mayonnaise containing nanoemulsions showed more pleasant results compared with mayonnaise containing pure garlic extract, this could be due to the controlled release of bioactive ingredients in nanoemulsions. The mayonnaise containing nanoemulsions also obtained higher scores in organoleptic tests due to encapsulating and controlled release compared with samples that have pure garlic extract.

## AUTHOR CONTRIBUTIONS

**Hamed Hassanzadeh:** Data curation (equal); investigation (equal); writing – original draft (equal). **Mahshid Rahbari:** Investigation (equal); software (equal). **Yaseen Galali:** Validation (equal). **Babak Ghanbarzadeh:** Supervision (equal). **Mohamadyar Hosseini**: Final edition; Statistical analysis.

## FUNDING INFORMATION

The author(s) received no financial support for the research, authorship, and/or publication of this article.

## CONFLICT OF INTEREST STATEMENT

We wish to confirm that there are no known conflicts of interest associated with this publication.

## Data Availability

The data that support the findings of this study are available from the corresponding author, upon reasonable request.
